# Supplementary material for: Comparative genomics provides new insights into the diversity, physiology, and sexuality of the only industrially exploited tremellomycete: Phaffia rhodozyma
Source: BMC Genomics. 2016 Nov 9;17:901. doi: 10.1186/s12864-016-3244-7 (PMC5103461; doi:10.1186/s12864-016-3244-7)
Supplement: Additional file 6: — List of orphan genes with links to PFAM (related to Additional file 1: Table S1). (ZIP 1428 kb) [file 12864_2016_3244_MOESM6_ESM.zip › PFAM_UNIQUE/G01806_P.html]

#                                                                            --- full sequence --- -------------- this domain -------------   hmm coord   ali coord   env coord
  
# target name        accession   tlen query name           accession   qlen   E-value  score  bias   #  of  c-Evalue  i-Evalue  score  bias  from    to  from    to  from    to  acc description of target
  
#------------------- ---------- ----- -------------------- ---------- ----- --------- ------ ----- --- --- --------- --------- ------ ----- ----- ----- ----- ----- ----- ----- ---- ---------------------
  
G01806\_P             -            422 DUF1469              PF07332.6    121     0.051   12.0   0.2   1   1   7.3e-05     0.087   11.2   0.2    70   100   228   258   196   264 0.88 - Protein of unknown function (DUF1469)  
G01806\_P             -            422 DUF3592              PF12158.3    148     0.063   11.7   0.8   1   2      0.13   3.9e+02   -0.6   0.8    37    69   164   194    73   226 0.68 - Protein of unknown function (DUF3592)  
G01806\_P             -            422 DUF3592              PF12158.3    148     0.063   11.7   0.8   2   2   9.3e-05      0.28    9.6   0.0     3    83   233   404   231   421 0.71 - Protein of unknown function (DUF3592)  
G01806\_P             -            422 FixQ                 PF05545.6     49     0.039   12.3   0.2   1   1   2.7e-05     0.082   11.3   0.2    10    36   232   258   230   265 0.85 - Cbb3-type cytochrome oxidase component FixQ
